# Neural Differentiation of Embryonic Stem Cells *In Vitro*: A Road Map to Neurogenesis in the Embryo

**DOI:** 10.1371/journal.pone.0006286

**Published:** 2009-07-21

**Authors:** Elsa Abranches, Margarida Silva, Laurent Pradier, Herbert Schulz, Oliver Hummel, Domingos Henrique, Evguenia Bekman

**Affiliations:** 1 Instituto de Medicina Molecular, Faculdade de Medicina de Lisboa, Lisboa, Portugal; 2 Sanofi-Aventis, Centre de Recherche de Paris, Paris, France; 3 Max-Delbrück-Center for Molecular Medicine (MDC) Berlin-Buch, Berlin, Germany; McMaster University, Canada

## Abstract

**Background:**

The *in vitro* generation of neurons from embryonic stem (ES) cells is a promising approach to produce cells suitable for neural tissue repair and cell-based replacement therapies of the nervous system. Available methods to promote ES cell differentiation towards neural lineages attempt to replicate, in different ways, the multistep process of embryonic neural development. However, to achieve this aim in an efficient and reproducible way, a better knowledge of the cellular and molecular events that are involved in the process, from the initial specification of neuroepithelial progenitors to their terminal differentiation into neurons and glial cells, is required.

**Methodology/Principal Findings:**

In this work, we characterize the main stages and transitions that occur when ES cells are driven into a neural fate, using an adherent monolayer culture system. We established improved conditions to routinely produce highly homogeneous cultures of neuroepithelial progenitors, which organize into neural tube-like rosettes when they acquire competence for neuronal production. Within rosettes, neuroepithelial progenitors display morphological and functional characteristics of their embryonic counterparts, namely, apico-basal polarity, active Notch signalling, and proper timing of production of neurons and glia. In order to characterize the global gene activity correlated with each particular stage of neural development, the full transcriptome of different cell populations that arise during the *in vitro* differentiation protocol was determined by microarray analysis. By using embryo-oriented criteria to cluster the differentially expressed genes, we define five gene expression signatures that correlate with successive stages in the path from ES cells to neurons. These include a gene signature for a primitive ectoderm-like stage that appears after ES cells enter differentiation, and three gene signatures for subsequent stages of neural progenitor development, from an early stage that follows neural induction to a final stage preceding terminal differentiation.

**Conclusions/Significance:**

Overall, our work confirms and extends the cellular and molecular parallels between monolayer ES cell neural differentiation and embryonic neural development, revealing in addition novel aspects of the genetic network underlying the multistep process that leads from uncommitted cells to differentiated neurons.

## Introduction

Neural induction in vertebrate embryos was first described by Mangold and Spemann in 1924 [1] and results in the establishment of a neuroectodermal *primordium* from where the nervous system will arise. The molecular signals involved in this crucial event are not yet totally elucidated but it is known that FGF and WNT signalling are required, together with inhibition of BMP signalling activity [2], [3]. In the mouse embryo, the initial population of specified neuroepithelial progenitors (NPs) is known to express various pan-neural genes, like *sox1* and *sox2* [4], [5]. These NPs will then acquire competence to produce neurons when they become part of the closing neural tube during neurulation, in a process that involves retinoid signalling from adjacent somites and the activity of proneural genes [6].

The embryonic neural tube is composed by a pseudostratified layer of neuroepithelial cells with a clear apico-basal polarity. The apical domain of these cells is located at the luminal surface and is delineated by the presence of apical protein complexes, like the PAR polarity complex [7], as well as by the presence of junctional structures, where N-cadherin and β-catenin accumulate [8]. Centrosomes also localize apically in neuroepithelial cells, which enter mitosis close to the luminal surface due to the characteristic interkinetic nuclear movement (INM) [9]. This particular organization of the neural tube is important for the coordinated production of neurons and glia. Neighbouring neuroepithelial cells signal to each other through Delta/Jagged ligands and Notch receptors, in a process that maintains a population of proliferating NPs and coordinates the timely production of neurons throughout embryonic development (reviewed in [10], [11]). This unique architecture of the embryonic neural tube has transient character and disappears perinatally to give way to definitive CNS structures like the brain and spinal cord.

Several approaches have been used to achieve *in vitro* neural differentiation starting from embryonic stem (ES) cells, aimed at generating regionally specified neural progenitors and/or differentiated neuronal and glial subtypes. All these methods try to recapitulate, in different ways, the multistep process of neural development that occurs in the embryo, from neural induction to the terminal differentiation of neurons and glial cells. This was initially achieved through embryoid body (EB) formation in the presence of retinoic acid [12] or, alternatively, by co-culture of ES cells with stroma/conditioned medium [13], [14]. However, as ES cells are pluripotential and readily differentiate into almost any cell type, the efficiency of neural conversion is limited and lineage selection is usually needed to ensure homogeneity of the differentiated population [15]. A simpler way to reconstitute neural commitment *in vitro* and achieve efficient neuronal production relies upon monolayer differentiation of ES cells, a method developed by Ying and co-workers [16]. In this method, ES cells are cultured in defined serum- and feeder-free conditions, in the absence of BMP signals that are known to inhibit neural fate. In these conditions, ES cells undergo neural commitment through a “autocrine” induction mechanism, where FGF signalling plays a pivotal role, as it does in the embryo [17], [18]. This method results in a more efficient neural commitment and differentiation, which likely results from a better mimicry of the events that occur in the embryo. However, a detailed characterization of the cellular and molecular steps involved in promoting ES cell differentiation towards neural lineages is required, not only to enhance our understanding of neurodevelopmental mechanisms but also to develop more rational ES cell-based strategies for treating traumatic injuries and neurodegenerative diseases affecting the human nervous system.

In this work, we describe various aspects of the process that leads from ES cells to differentiated neurons in monolayer cultures. Using improved conditions, we routinely obtain highly homogeneous cultures of NPs that maintain morphological and functional characteristics of their embryonic counterparts, namely apico-basal polarity, active Notch signalling, and proper timing of production of neurons and glia. We show that the transition to neuronal production is accompanied by the organization of NPs into neural tube-like rosettes, where these cells divide and give rise to neurons. Furthermore, we have characterized the global gene expression changes that occur along the path to neural differentiation, from ES cells to neurogenic rosettes. Our results confirm and extend at the molecular level the parallels with embryonic neural development, revealing in addition novel aspects of the genetic network underlying the multistep process that leads from uncommitted cells to differentiated neurons.

## Results

### Improved generation of NPs from ES cells in defined serum-free media

**Figure 1:**
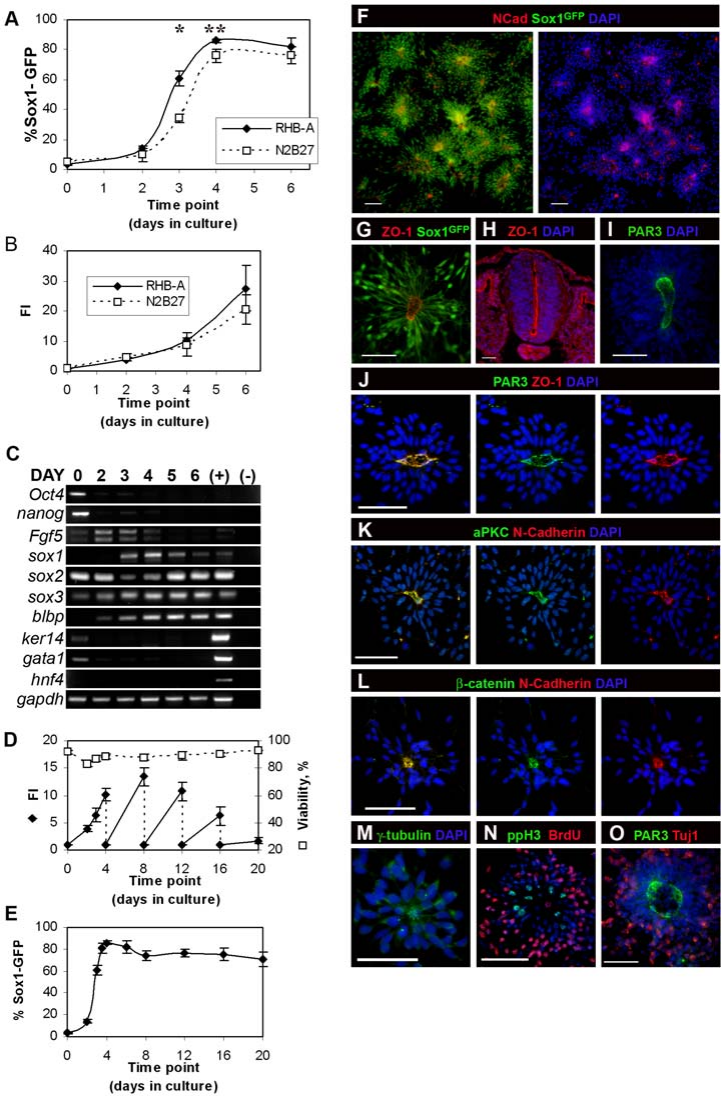
ES-cell derived NPs culture analysis. A) Percentage of GFP^+^ cells in monolayer cultures grown for 6 days without replating in RHB-A and N2B27 media (* p-value = 0.005; ** p-value = 0.052). B) Fold increase (FI) for monolayer cultures grown for 6 days in RHB-A and N2B27 media. C) Semi-quantitative RT-PCR analysis for selected markers of pluripotency and lineage commitment in day 0–6 RHB-A cultures; mRNA from E10.5 mouse embryos was used as positive control. D) FI (filled squares) and viability (open squares) for RHB-A cultures maintained for 20 days in culture and replated every 4 days at the same initial cell density. E) Percentage of Sox1-GFP^+^ cells along 20 days in culture in RHB-A, with replating every 4 days. In all graphs data are means±SEM from at least three independent experiments. F) After replating in laminin (day 5), Sox1-GFP^+^ cells organize in rosettes, with N-Cadherin (in red) present at the centre of these cell clusters. G) ZO-1 accumulates in the cell processes that coalesce at the centre of rosettes, like it does in the apical domain of NPs in the embryonic neural tube (H). I) Anti-PAR3 immunostaining reveals well-defined “apical” domains at the centre of rosettes, where it co-localizes with ZO-1 (J). K) aPKC, another known apical marker is also present at the centre of rosettes and co-localizes with N-Cadherin. L) Adherent junctions' components, ß-catenin and N-Cadherin, co-localize at the central, apical region of rosettes. M) Anti-γ-tubulin staining (in green) shows “apically” localized centrosomes. N) Mitotic figures (ppH3) are localized centrally in rosettes while S-phase nuclei (BrdU) are located at the periphery. O) Differentiating Tuj1^+^ neurons accumulate at the periphery of rosettes. Nuclei counterstained with DAPI (blue). Scale bar: 50 µm.

Commitment of undifferentiated ES cells to neural fate can be achieved with high efficiency in feeder-free adherent monocultures, using the serum-free medium N2B27 [16]. In these conditions, when *Sox1-GFP* knock-in (46C) ES cells were used, Ying and co-workers reported that cultures with more than 80% of NPs (Sox1-GFP^+^) can be obtained [16]. Using the same ES cell line, we carried out a comparative study of neural commitment in N2B27 and RHB-A (StemCellSciences Inc., UK), a new N2B27-based neural differentiation medium. We monitored cellular growth, the emergence of Sox1-GFP^+^ NPs and the appearance of various cell-specific markers in these cultures. Our results show that commitment to neural fate in RHB-A occurs faster and produces a higher percentage of Sox1-GFP^+^ NPs, when compared to N2B27 (Fig. 1A). For instance, three days after ES cell plating in RHB-A, more than 60% of cultured cells are Sox1-GFP^+^ NPs, while only about 40% of cells became Sox1-GFP^+^ in N2B27 (p-value = 0.005). The percentage of Sox1-GFP^+^ NPs in the total population reaches a peak at day 4 in both media, with consistently higher levels in RHB-A (p-value = 0.052). Further culturing for 2 more days results in a sharp increase in the total number of cells (Fig. 1B), but without changes in the percentage of Sox1-GFP^+^ NPs (Fig. 1A). This suggests that, from day 4 onwards, a “transit-amplifying” population of Sox1-GFP^+^ NPs is established and that induction of new NPs contributes little to the growth of this population.

RT-PCR analysis confirms that the switch from ES identity (*Oct4* ^+^, *nanog* ^+^, *sox2* ^+^, *sox1* ^−^, *sox3* ^−^) to that of NPs (*sox1^+^,2^+^,3* ^+^, *blbp* ^+^,*Oct4* ^−^ and *nanog* ^−^) seems to be complete by day 4 (Fig. 1C). Before this, cells pass initially through a primitive ectoderm (PE) stage, as shown by the expression of *Fgf5* [19], [20], preceding the appearance of NP markers at day 3. In contrast, markers for endodermal (*hnf4*, *gata1*) and epidermal (*ker14*) lineages are rapidly down-regulated during monolayer differentiation (Fig. 1C).

Based on these results, we chose to replate day 4 NPs onto a laminin substrate in the same RHB-A medium, to test their neural differentiation potential. The cultures were maintained until day 20, being replated every 4^th^ day. In these conditions, cell viability remains high (above 90%), although the proliferation rate (shown as fold increase–FI) decreases along time (Fig. 1D). The percentage of Sox1-GFP^+^ NPs in culture also decreases, stabilizing above 70% around day 8 (Fig. 1E).

### NPs show proper apico-basal polarity *in vitro* and undergo INM

After replating, we observed that cells grow in tightly packed monolayers resembling thick epithelial sheets. However, the distribution of Sox1-GFP^+^ cells is not uniform in these sheets, being organized in clusters to form rosette-like structures (Fig. 1F). In these clusters, Sox1-GFP^+^ NPs express the known apical markers of neuroepithelial cells, N-cadherin and ZO-1, which are localized at the centre of rosettes (Fig. 1F,G). This suggests that NPs within these structures are organized with their apical domains coalescing to form a central lumen, like in the embryonic neural tube (Fig. 1H). This organisation is confirmed by the co-localization of other known neuroepithelial apical markers at the centre of rosettes, like PAR3 (Fig. 1I,J), aPKC (Fig. 1K), β-catenin (Fig. 1L), Numb, Afadin and Occludin (not shown). Furthermore, centrosomes are located close to the central region of the rosettes (Fig. 1M), where mitotic (ppH3^+^) nuclei are also detected (Fig. 1N). In contrast, S-phase nuclei lie at the periphery of rosettes, as shown by short pulses of BrdU labelling (Fig. 1N). This suggests that the nuclei of NPs within rosettes reproduce the characteristic INM shown by NPs in the embryonic neural tube [9]. To confirm this, we carried out time-lapse imaging of ES cell-derived neural rosettes in culture, revealing that NPs do indeed undergo nuclear movements coupled with the cell cycle, like embryonic NPs (Movie S1). Finally, newborn neurons (Tuj1^+^) localize outside or at the periphery of rosettes (Fig. 1O), resembling also the embryonic neural tube where neurons accumulate outside of the ventricular proliferative zone. Together, these observations reveal that rosettes are remarkably organized like embryonic neural tubes, with ES cell-derived NPs linked by junctional structures at their apical surface and engaged on neurogenesis. This led us to explore whether rosette-like cultures may have other structural and functional similarities with the embryonic neural tube.

### Notch pathway is active in rosette cultures

**Figure 2:**
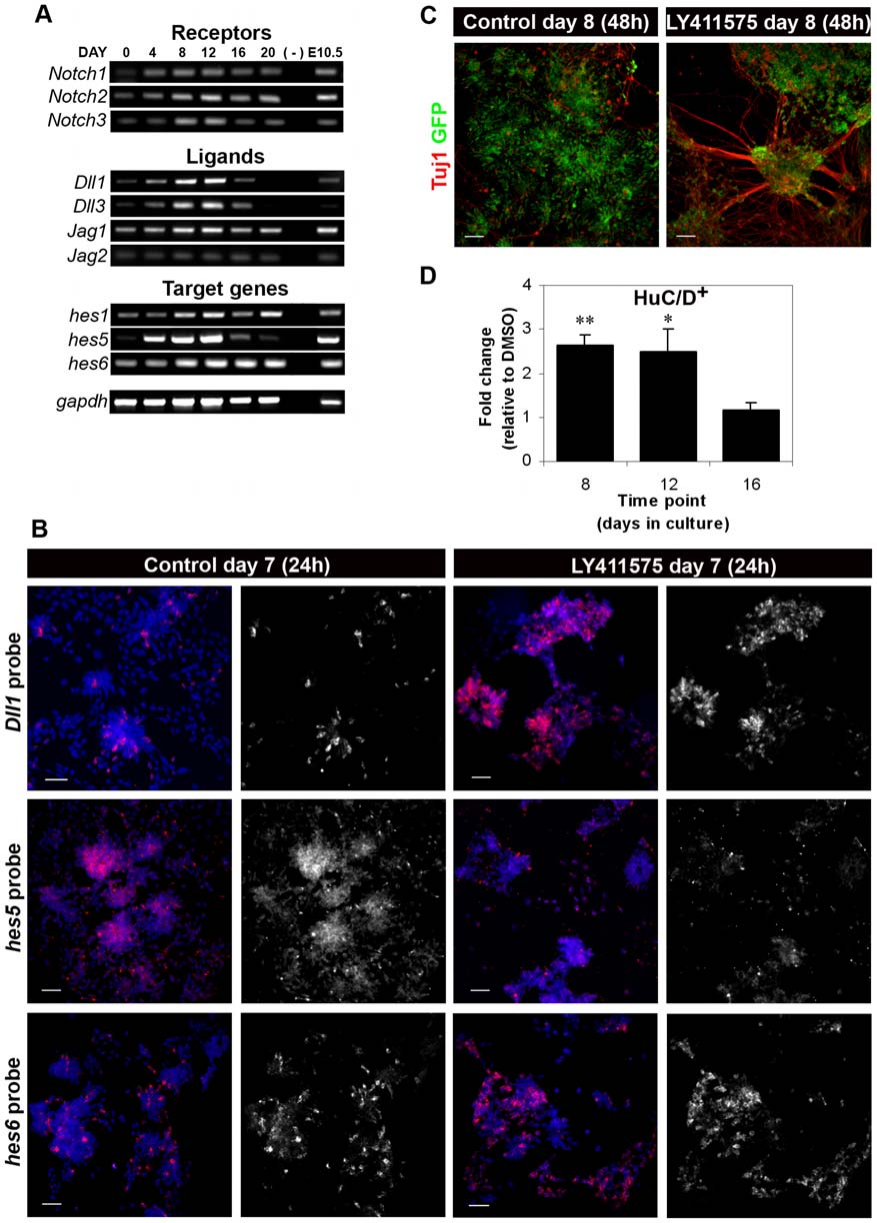
Chemical inhibition of the Notch activity by γ-secretase inhibitor LY411575. A) Expression of Notch pathway genes during monolayer ES cell differentiation, from day 0 to day 20, by RT-PCR analysis. mRNA from E10.5 mouse embryos was used as control. B) Detection by ISH of *Dll1*, *hes5* and *hes6* transcripts in control (DMSO-treated) and LY411575-treated rosette cultures. Treatment was done in day 6 cultures for 24 hours. Nuclei counterstained with DAPI. C) After 48 h of LY411575 treatment, starting at day 6, massive neuronal differentiation is observed by Tuj1 immunostaining. D) Notch inhibition with LY411575 at day 8 or 12 of the monolayer protocol results in increased neuronal production, detected by HuC/D imunostaining. No change was detected when inhibition was done in day 16 rosettes. Bars in D represent SEM for the minimum of three independent experiments. * p-value = 0.025; ** p-value = 0.002. Scale bars in B,C: 50 µm.

In the embryonic neuroepithelium, the Notch pathway controls the rate at which proliferating NPs commit to differentiation. When Notch activity is inhibited, precocious neuronal differentiation is usually observed (reviewed in [21]). To test whether Notch signalling is involved in maintaining NPs in cultured neuroepithelial rosettes, as it happens in the embryonic neuroepithelium, we first analysed the expression of various genes known to mediate Notch activity (Fig. 2). RT-PCR data show that *Notch1*, *2* and *3* are expressed in monolayer cultures, together with various *Delta-like* and *Jagged* genes, as well as *hes* genes known to be involved in embryonic neural development (Fig. 2A). Analysis by *in situ* hybridization (ISH) reveals that *hes5*, the main Notch target gene in embryonic NPs [22], is broadly expressed in neuroepithelial rosettes, while *Dll1* and *hes6*, which are normally expressed in newborn neurons [23], [24], show a more scattered expression, consistent with being transcribed in rosette cells singled out for differentiation (Fig. 2B, left panels). To evaluate the functional role of Notch signalling in rosette cultures, its activity was inhibited by treatment with the γ-secretase inhibitor LY411575 [25], resulting in a strong reduction of *hes5* expression and the concomitant increase in *Dll1* and *hes6* expression (Fig. 2B, right panels). These results confirm the efficacy of Notch inhibition and show that rosette progenitors embark on neuronal differentiation in the absence of Notch activity. Indeed, LY411575-treated cultures reveal a significant increase both in Tuj1^+^ (Fig. 2C) and HuC/D^+^ (Fig. 2D) neurons, accompanied by striking morphological changes: after 48 h of Notch inhibition, rosette structures disappear and give way to large rounded ganglion-like clusters made up by Tuj1^+^ and HuC/D^+^ differentiating neurons, with extensive neurite outgrowths. Quantification of the number of HuC/D^+^ differentiating neurons reveals that the neurogenic effect due to Notch inhibition is more pronounced in day 8 cultures (n = 3, p-value = 0.002), while day 16 cultures show no increase of neuronal production (Fig. 2D). However, Notch receptors and ligands are still expressed at day 16, albeit at lower levels, making it unlikely that the lack of neurogenic effects is due to the absence of some components of the pathway. An alternative explanation is that, by day 16, NPs have lost most of their neurogenic potential and have switched their competence to gliogenic, as it has been previously described to happen during embryonic neural development and in cultures of isolated cortical NPs [26], [27].

### NPs have both neurogenic and gliogenic potential *in vitro*

**Figure 3:**
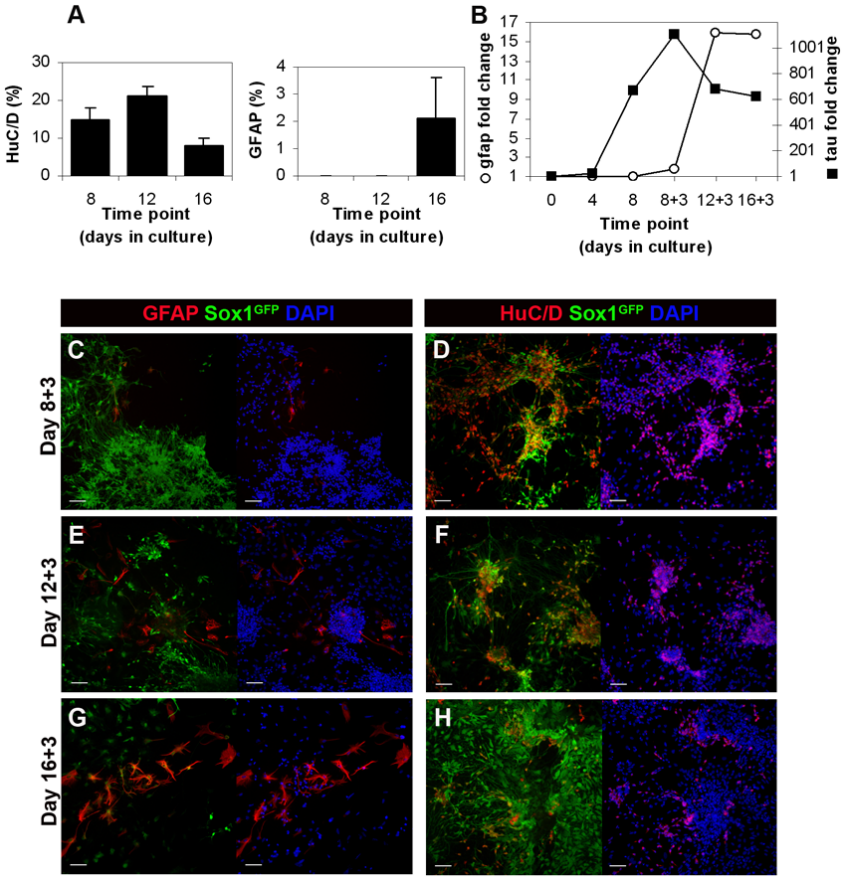
Timing of production of neurons and glia in rosette cultures. A) Percentage of HuC/D^+^ and GFAP^+^ cells in rosette cultures, relative to the total number of cells in culture. A decrease in neuronal production is observed at day 16, concomitant with an increase of glial cells. B) Semi-quantitative RT-PCR data showing fold change of expression (relative to day 0) for neuronal (*tau*) and glial (*gfap*) markers at successive timepoints of rosette cultures. Data normalized to *gapdh*. C–H) Rosette cultures at day 8+3, 12+3 and 16+3, labelled with anti-HuC/D and anti-GFAP antibodies to visualize neurons and glial cells, respectively. Few GFAP^+^ cells appear in day 8+3 cultures (C), with the number increasing at day 12+3 (E) and 16+3 (G). In contrast, a decrease in the number of HuC/D^+^ neurons is detected at day 16+3 (H). Nuclei counterstained with DAPI (blue). Scale bars: 50 µm.

To test whether NPs in rosette cultures undergo a temporal switch from early, neuron-producing, to late, glia-producing progenitors, we quantified the production of neurons and glia throughout the monolayer differentiation protocol. We found that the number of HuC/D^+^ neurons in rosette cultures increases up to day 12 and starts to decrease by day 16 (Fig. 3A). On the contrary, GFAP^+^ glial cells can only be detected from day 16 on, after the third replating, albeit still in reduced numbers (Fig. 3A). We reasoned that 4 days of culture after replating might not be sufficient to allow for glial differentiation and appearance of GFAP immunoreactivity. We therefore extended cultures for 3 additional days without replating (days 8+3, 12+3 and 16+3). In these conditions, we could detect scattered GFAP^+^ cells as early as day 8+3, although still in reduced numbers (no more than 3 cells per coverslip, Fig. 3B,C), in striking contrast with the number of HuC/D^+^ differentiating neurons generated at the same time (Fig. 3D). In day 12+3 cultures, GFAP^+^ cells can be detected consistently (Fig. 3E), though they still appear in much lower numbers than HuC/D^+^ neurons (Fig. 3F). The maximum number of GFAP^+^ cells occurs at day 16+3 (Fig. 3G), in contrast to that of HuC/D^+^ cells which peak at day 8+3 (Fig. 3H). Together, these results reveal that the neurogenic potential of rosette cultures decreases with time, with GFAP^+^ glial cells appearing consistently after the peak of neuronal production (Fig. 3B), indicating that a switch of progenitor identity, from neurogenic to gliogenic, occurs in these cultures.

### Neural stem cells are present in monolayer cultures

**Figure 4:**
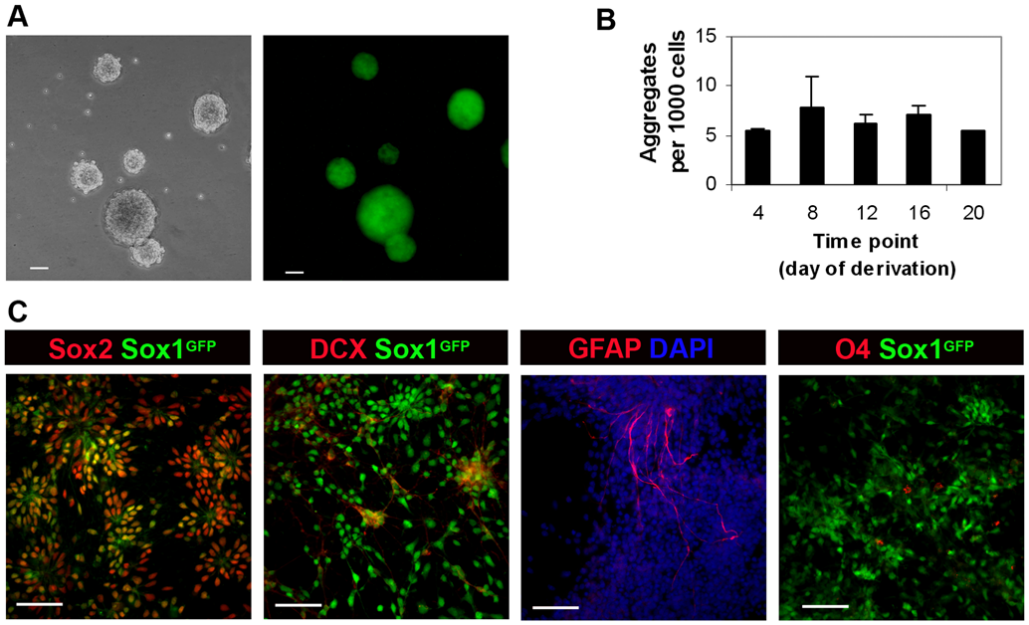
NS cell potential of the *in vitro* neuroepithelial rosette cultures. A) Floating aggregates of NS cells derived from day 4 monolayer cultures (Sox1-GFP 46C cells; phase contrast and GFP fluorescence images). B) Efficiency of derivation of NS cells-derived floating aggregates from several rosette cultures time points (days 4, 8, 12, 16 and 20), expressed as number of aggregates formed per 1000 cells. Bars represent SEM for 3 independent experiments. C) Floating aggregates of NS cells (derived from day 4 monolayer cultures of 46C) were left to attach for 4 days onto laminin substrate and stained for Sox2 (NP marker), Doublecortin (DCX, neuronal marker), GFAP (glia) and O4 (oligodendrocytes). Scale bars: 50 µm.

It is known that both embryonic neural tissue and certain regions of the adult vertebrate CNS contain a resident population of progenitor/stem cells [28]. Recent work [29], [30] established conditions for the isolation and clonogenic *in vitro* propagation of neural stem (NS) cells derived either from ES cells or from embryonic and adult neural tissue. In the present work, using the same experimental conditions, we were able to derive floating aggregates of NS cells from all stages of the *in vitro* neuroepithelial rosette cultures (days 4, 8, 12, 16 and 20) with similar efficiencies (Fig. 4A,B). When these aggregates are plated *en bloc* onto laminin substrate, without dissociation, cells migrate out and form neuroepithelial rosettes where all cells are positive for the NS cells markers Sox2 (Fig. 4C) and Nestin (not shown). After several days of culture, these cells develop very long cellular projections similar to those of radial glia (Fig. 4C and data not shown) and are able to differentiate into the all three neural lineages (Fig. 4C), a feature that fits well with the characteristics of NS cells. The constant presence of these cells, both in less proliferative day 20 monolayer cultures as well as in younger day 4 cultures (Fig. 4B), indicates that the floating aggregates are derived from a resident stem cell population, present in neuroepithelial rosette cultures at all time points studied. This, in turn, provides additional evidence for the neuroepithelial identity of these cultures.

### Transcriptional profiling of *in vitro* neural commitment

**Figure 5:**
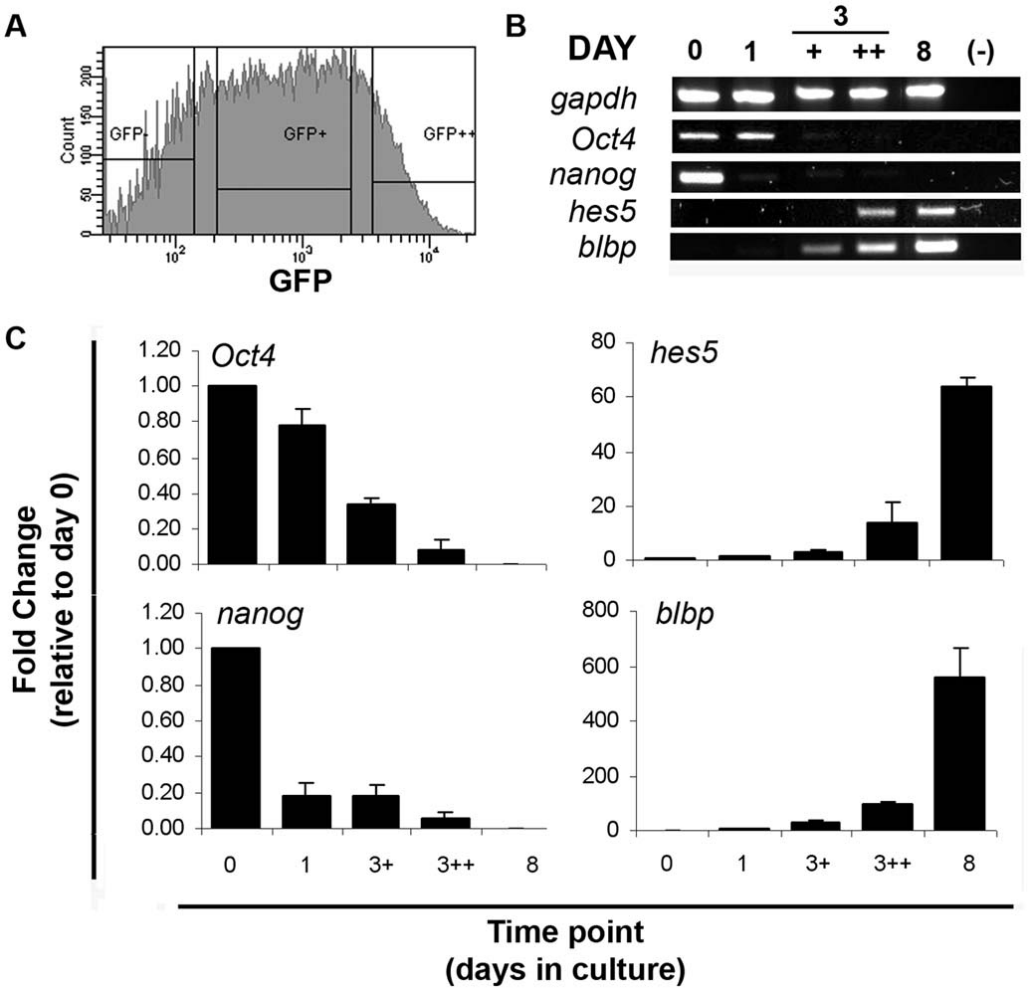
Validation of microarrays results. A) Histogram of sorted Sox1-GFP populations from day 3 monolayers. GFP negative (GFP-) cells were discarded, while two GFP positive populations were collected individually, according to their levels of GFP expression (GFP+ and GFP++). B) RT-PCR analysis of RNA samples collected for microarray analysis for the genes *Oct4*, *nanog*, *hes5*, and *blbp*. C) Fold changes, relative to day 0, obtained from Affymetrix profiling for the genes *Oct4*, *nanog*, *hes5*, and *blbp*.

The results described above indicate that neuroepithelial rosette cultures recapitulate several aspects of embryonic neural tube development. In contrast to the scarcity and complexity of cells from early stages of mammalian embryos, these cultures can provide large and highly homogeneous populations of cells at various stages of neural development, with the additional advantage of obtaining homogeneous populations of Sox1-GFP^+^ NPs by FACS sorting. This creates a unique opportunity to characterize the transcriptional programs active at various phases of neural commitment and differentiation, from which it might be possible to predict the molecular pathways regulating these processes. With this purpose, global gene expression profiling using Affymetrix microarrays (Mouse Genome 430 Version 2.0) was performed at several stages of the monolayer rosette cultures: day 0 (undifferentiated ES cells), day 1, day 3 and day 8. At day 1, ES cells have entered differentiation and our aim was to obtain a gene signature for a population of primitive ectoderm-like cells that is likely to be present, as marked by the up-regulation of *Fgf5* expression and down-regulation of *nanog* (Fig. S1). At day 3, a sharp up-regulation of *sox1* is detected by RT-PCR (Fig. 1C), probably reflecting the emergence of an initial population of NPs after neural induction. To characterize the transcriptional program active in these early NPs, we chose to purify Sox1-GFP^+^ cells at day 3 by FACS sorting, resulting in two sub-populations according to the levels of GFP expression (GFP+ and GFP++, Fig. 5A). Our prediction was that cells with lower levels of GFP might be at an earlier stage of NP development and that, by separating the two sub-populations of NPs, one could pull out genes associated with the earliest NPs state. Finally, at day 8, NPs are organized in rosettes and already engaged on neurogenesis, in a stage likely to be equivalent to NPs from the embryonic neural tube, after the onset of neuronal differentiation [55].

At least three independent RNA preparations from each of the selected time points were processed and hybridized on the arrays. Previous validation of these samples was done by analyzing the expression of *Oct4*, *nanog*, *hes5* and *blbp* by semi-quantitative RT-PCR (Fig. 5B). As expected, expression of ES cell genes, *Oct4* and *nanog*, decrease throughout the differentiation process and are no longer detected on day 3. In contrast, expression of NP markers, *hes5* and *blbp*, can only be detected at day 3, increasing significantly at day 8. This pattern of expression was also observed in the microarray profiling (Fig. 5C).

**Figure 6:**
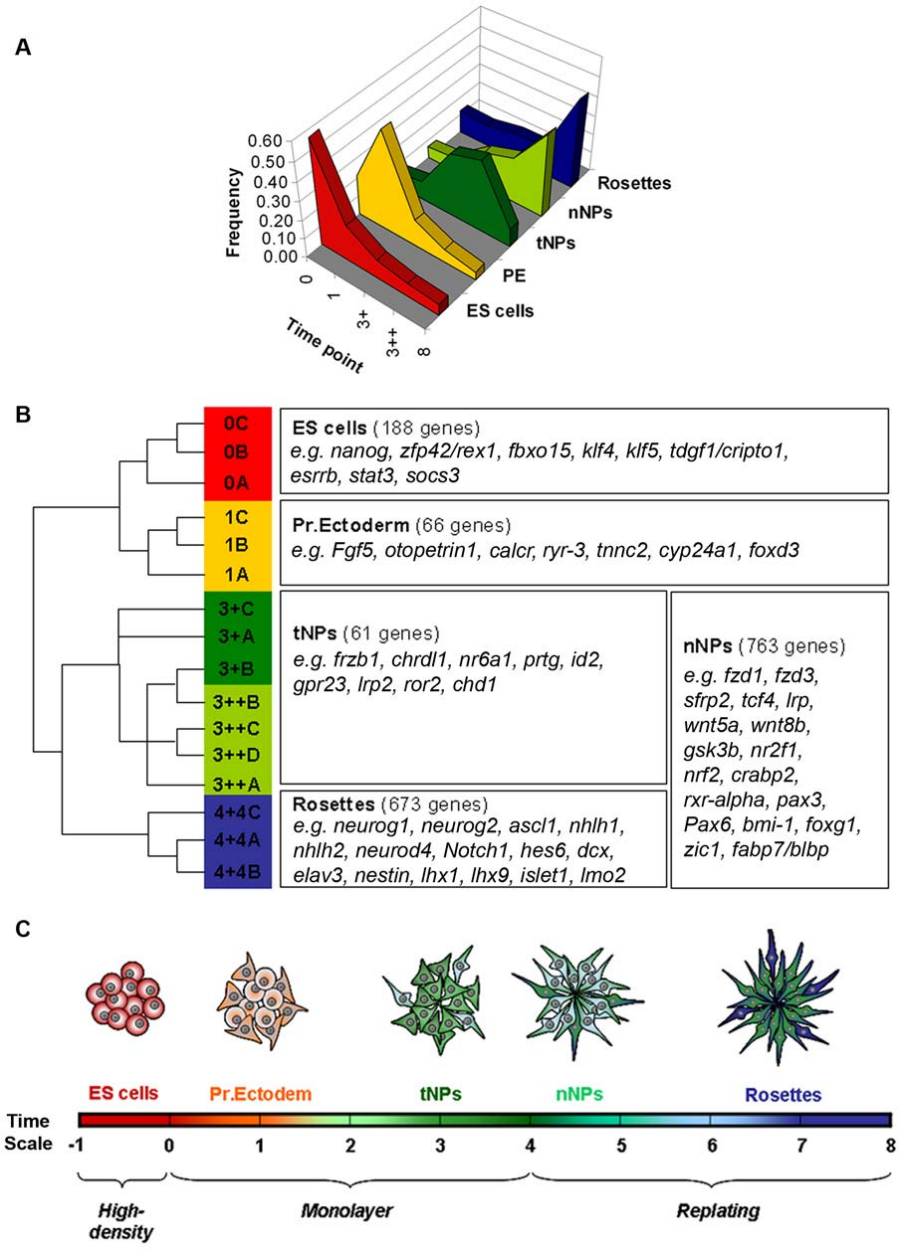
Clustering analysis of differentially expressed genes. A) Frequency distribution of the expression levels of the genes belonging to the five defined groups. B) Dendogram of the relationship of expression of genes belonging to each group (with biological replicates being represented by the letters A, B, C and D) and examples of genes that are present in the five defined groups. C) Schematic representation of the successive cellular states that occur along the path to neural differentiation (see text for definitions of stages).

The microarray data were normalized by the log scale robust multi-array analysis [31] and an ANOVA FDR-value of 10^−3^ (p-value<2.10^−4^) was used to identify and restrict the number of differentially expressed probe sets to 9456 (Table S1), which correspond to 6563 unique genes. Further analysis of the differentially expressed genes involved their distribution into specific groups, according to the variations in their expression throughout differentiation (Fig. 6 and Table S1). A first group was defined as including genes whose expression peaks at day 0 and is downregulated at all other time points. This includes known pluripotency genes like *nanog*, *rex1* and *fbxo15*, confirming the ES cell identity of the initial population at day 0. A second group includes genes with a peak of expression at day 1 and might identify a transient PE population as indicated by the presence of *Fgf5* in this group [19], [20]. A third group includes genes that are up-regulated at day 3 but down-regulated in day 8 rosettes, and might characterize a transient population of Sox1-GFP^+^ NPs (tNPs) that emerge after neural induction. These progenitors will then evolve into neurogenic progenitors (nNPs) competent to initiate neuronal production, identified by a fourth group containing genes that start to be up-regulated at day 3 but continue to be expressed at similar or higher levels in day 8 rosettes. Finally, a fifth group is composed by genes that are only up-regulated in day 8 rosettes and includes genes characteristic of progenitors in the final phase of commitment to differentiation, like the proneural genes *ascl1*, *neuroG1* and *neuroG2* [32], as well as genes known to be expressed in early differentiating neurons, like *doublecortin* and *hu/elav* [33], [34].

This distribution of transcriptional profiles depicts, at the molecular level, the successive cellular states that occur along the path to neural differentiation, allowing the identification of gene signatures for each of these states and a better definition of the transitions between them.

## Discussion

In this work, we characterize at the cellular and molecular level the processes of neural commitment and differentiation that occur when mouse ES cells are driven into a neural fate, using an improved adherent monolayer protocol [16], [35]. We show that NPs derived from ES cells organize themselves into rosette-like structures, with an apico-basal distribution of polarity proteins similar to that described for neuroepithelial cells in the embryonic neural tube [7], [8]. In addition, ES cell-derived rosette NPs display the characteristic cell cycle-related INM of the embryonic neuroepithelium. We also show that Notch signalling is active in neuroepithelial rosettes and controls the timely production of neurons from ES cell-derived NPs. The intrinsically controlled sequential generation of neurons and glial cells seems to be also preserved in ES cell-derived NPs. Altogether, these results demonstrate that the *in vitro* generation of neural cells from ES cells, using the monolayer protocol, closely mimics the process of embryonic neural development. Global gene expression analysis, at successive steps of the process that leads ES cells to neurons, provides further support for the similarities between the ES cell-derived rosette culture system and embryonic neural tube development, revealing in addition novel candidate genes that might regulate the processes of neural commitment and differentiation.

### Neuroepithelial rosettes as *in vitro* counterparts of embryonic neural tube

Several methods have been described to achieve neural differentiation of ES cells *in vitro* (reviewed in [36], [37]), including the treatment of cell aggregates (EBs) with retinoic acid [12] or the co-culture of ES cells with stromal cells that produce uncharacterized neural-inducing factors [14]. The concept of neural induction as a default pathway for differentiation in early vertebrate embryos [2] led to the development of a simple adherent monolayer culture system where ES cells are driven by autocrine signalling into a neural fate, using a defined serum-free media (N2B27). In these conditions, endogenous FGF and Notch signalling seem necessary for ES cells to enter the neural pathway [17], [18], [38], together with the production of endogenous inhibitors of BMP signalling [16]. Using this monolayer protocol, one can routinely obtain an enriched population of NPs (up to 80%) after 4–6 days in culture, although the presence of “contaminating” cells (undifferentiated ES cells and large flatten non-neural differentiating cells) is still observed, probably due to some remaining endogenous BMP signalling. In this work, we report that the use of a new N2B27-derived medium (RHB-A) allows a faster and more efficient production of NPs from ES cells in monolayer culture (Fig. 1A), resulting in highly homogeneous populations of NPs (up to 90%) that can subsequently differentiate into neurons, astrocytes and oligodendrocytes. The observation in the reduction of large flatten non-neural cells in RHB-A cultures correlates with the observed decrease of BMP4 expression (Fig. S2), along with an increase in the expression of known BMP-antagonists (e.g. Chordin-like1, Follistatin), in contrast to what has been reported for N2B27 cultures [16].

The transition from ES cells to NPs in RHB-A monolayer cultures is accompanied by the organization of these NPs into characteristic rosette-like structures, in a process that resembles neural tube formation in the embryo. The formation of similar rosettes has been described in other *in vitro* models of neural differentiation from ES cells [39], [40], suggesting that this is a common behaviour of NPs, associated with their epithelial character. We have studied in detail the organization of these rosettes and show that several proteins normally present in the apical domain of embryonic neuroepithelial cells, like N-Cadherin, ß-catenin, Par3/aPKC and Numb, are localized close to the luminal centre of rosettes, revealing that ES cell-derived NPs are able to acquire a proper apico-basal organization, despite being cultured in a 2D environment. In addition, we show that NPs within rosettes display the characteristic INM observed in the embryonic neuroepithelium, with progenitors entering mitosis when their nuclei are closer to the luminal surface of rosettes. We also noted that differentiating neurons loose contact with the centre of rosettes and migrate to their periphery. Altogether, these findings reveal that NPs in culture are able to self-organize into neural tube-like structures, thus recapitulating the cellular interactions that regulate the process of neuronal production.

Notch activity is a major player in this process (reviewed in [11], [21], [41]) and the parallel between embryonic neurogenesis and *in vitro* neural differentiation of ES cells is reinforced by the similar dependence on Notch signalling to maintain a population of NPs engaged on neuronal production. Indeed, we show that Notch signalling is active in ES cell derived neuroepithelial rosettes and that chemical inhibition of Notch activity results in massive neuronal differentiation of rosette NPs, similarly to what has been described during CNS embryonic development (reviewed in [11], [21]). Interestingly, this drift to neuronal differentiation in the absence of Notch activity is no longer seen on day 16 cultures, suggesting that neuronal competence decreases with time. This is confirmed by the striking reduction in the number of neurons generated in later cultures, concomitant with an increase in the generation of GFAP^+^ glial cells, revealing a switch from neurogenic to gliogenic NPs in late monolayer cultures. This switch coincides with the disappearance of rosettes from the cultures: while ES cell-derived NPs grow exclusively in the form of rosettes up to day 12, few rosettes are still present at day 16 as neuronal production is significantly decreased and gliogenesis increases. These observations indicate that the intrinsic temporal regulation of neurogenic *vs.* gliogenic differentiation, characteristic of embryonic neural tube, is conserved in ES cell-derived rosette cultures. How this temporal regulation occurs *in vitro* is still unclear but must be independent of extrinsic cues, as previously reported for embryonic NPs [26], [27].

Altogether, our data extend the previous characterization of the monolayer protocol as an efficient and reproducible method to drive ES cells into a neural fate and provide further evidence that the steps involved in the *in vitro* acquisition of a neural fate closely mimic the events that happen during embryonic neural commitment and differentiation.

### Molecular mechanisms of *in vitro* mammalian neural development

The path from ES cells to a neural fate involves various transitions in the potential of the cells, starting with the conversion to a PE-like stage followed by the transition into neuroectoderm and establishment of a population of NPs that will gradually give rise, first, to differentiated neurons and, later, to glial cells. To characterize these cellular states at the molecular level and identify genes that might promote the transitions between successive stages, we have performed global transcriptome analysis of ES cells and their derivatives along the path to a neural fate. This resulted in the identification of a large set of genes (6.563) whose expression significantly changes throughout the monolayer neural differentiation protocol. Analysis of the data involved the clustering of these genes into five groups according to their expression profiles, which we correlated with diverse cell populations that emerge in the course to neural differentiation.

The first group comprises genes with a peak of expression at day 0 and that are rapidly down-regulated as ES cells loose their “stemness” character. This group includes known pluripotency markers of the ES cell state, like *nanog*, *zfp42/rex1*, *fbxo15*, *tdgf1/cripto1*, *socs3*, *esrrb*, *klf4* and *klf5*, and provides an ES cell signature that overlaps extensively with available data on ES cell specific transcripts [42], [43], [44]. Other known “stemness” genes like *Pou5f1/Oct4* and *sox2* are also strongly expressed in the starting population of ES cells but were excluded from our first gene group as their expression reappears in NPs (*sox2*) or takes longer to be down-regulated (*Pou5f1/Oct4*). Together, these data confirm the ES cell identity of the starting population of cells and could also serve to identify novel genes that might be important to maintain the ES cells status.

A second group includes the genes whose expression peaks at day 1 after ES cells have been plated in RHB-A, being subsequently down-regulated in NPs and neural rosettes. This group includes the PE marker *Fgf5* and might represent a gene signature for the PE-like stage that emerges after plating of ES cells in the absence of serum and leukaemia inhibitory factor (LIF). Until now, this stage has been characterized by the up-regulation of *Fgf5* expression and downregulation of *zfp42/rex1*, in a population still expressing *Oct4* [19], [45], [46], a pattern that is also observed in our data. Although epiblast stem cells (EpiSCs) reveal a similar expression profile [47], [59], the absence of markers of tripotency in our day 1 PE-like cells (e.g. *brachyury*, *otx2*, *gata4* and *gata6*), together with the down-regulation of most ES cell genes (included in group I), implies that this population is different from EpiSCs.

A survey of the 66 genes included in this PE-like group reveals the presence of 5 genes involved in calcium homeostasis (*calcR*, *ryr-3*, *otopetrin1*, *tnnc2* and *cyp24a1*) suggesting that calcium signalling plays an important role in the transition of pluripotent stem cells into ectodermal fates. Indeed, an increase in intracellular calcium has been reported to be important for neuralization of ectodermal cells [48]; hence, the activity of the 5 identified genes might contribute to regulate calcium signalling in PE-like cells transiting to a neural fate. It would, therefore, be interesting to test whether these genes, as well as other candidate PE markers present in this group, are expressed in the mouse embryonic PE and what function they have in this tissue.

A third group comprises genes that are up-regulated in NPs (Sox1-GFP^+^) at day 3 but down-regulated in day 8 neurogenic rosettes. This behaviour indicates that these genes might play a role in the establishment of the initial population of NPs, but are switched-off afterwards to allow the subsequent progression to differentiation. In the embryo, a gene that shows a similar behaviour is *sox1*, whose down-regulation in NPs seems to be required for their commitment to differentiation, due to its ability to block the neurogenesis-promoting activity of proneural factors [5], [49]. During ES cell differentiation, *sox1* expression also peaks in day 3 NPs but does not decrease enough in day 8 rosettes to be included in this group, due to the stringent criteria that was chosen. Still, this group contains various genes that are known to be transiently expressed in embryonic NPs and regulate their generation, like the BMP inhibitor *chdl1*, the Wnt modulator *frzb1* and the orphan nuclear receptor *nr6a1* [50], [51], [52], supporting the analogies with embryonic neural development. Of the 61 genes included in this group, 43 are known to be expressed in embryonic NPs (by screening publicly available databases), while there is incomplete or no available data on the expression of the remaining 18 genes. We therefore propose that this group provides a novel gene expression signature for a transient population of NPs (tNPs) that is established following neural induction but that it is not yet competent to enter neurogenesis. This absence of neurogenic competence correlates with the reduced expression of proneural genes in day 3 Sox1-GFP^+^ NPs and the lack of an increase in Notch activity, as measured by the expression of *Notch1* and its targets and effectors *hes5* and *hes6* (Fig. S3).

This proposed tNP population is also likely to exist in the mouse embryo but the small number and transient character of tNPs, together with the “dilution” effect due to the presence of several other cell types, has made difficult to pinpoint its existence. The genes we have identified here as markers of the tNP population may now allow the identification of similar progenitors in the mouse embryo and provide an entry point to dissect the genetic circuitry controlling this stage of neural development.

The fourth group comprises genes that are up-regulated in NPs but that, in contrast to tNP genes, continue to be expressed at similar or increased levels in day 8 neurogenic rosettes. Our strategy of separating NP genes into two groups with distinct expression profiles highlights, on one side, genes which are active only during progenitor specification (tNP group) and, on the other side, genes that might also be important for the next stage of NP development (nNP), when competence to enter neurogenesis is acquired. By analogy with embryonic neural development, nNPs are likely to be an *in vitro* counterpart of the progenitors present in the “transition zone” or “pre-neural tube”, located at the caudal open neural plate, rostral to the node but posterior to the level of the first somite [6], [53]. Indeed, a survey of nNP genes reveals that the transition to a proliferative neurogenic population observed in monolayer cultures is accompanied by a significant increase on the expression of genes connected to the retinoic acid signalling (e.g., *rxr-alpha*, *crabp2*, *nr2f1*, *nr2f2*) and Wnt pathway (e.g., *fzd1*, *fzd3*, *sfrp2*, *tcf4*, *wnt5a*, *wnt8b*, *gsk3ß*, *lrp1*), which are known to regulate NP competence *in vivo* [54], [55]. Together, our data provide an accurate gene signature for two populations of NPs (tNPs and nNPs), with a high degree of confidence that results from the fact that FACS-purified populations of NPs were used in our experiments.

A population of purified Sox1-GFP^+^ NPs has previously been studied in *Sox1^GFP^* transgenic mouse embryos with 15 genes being found to be preferentially expressed in embryonic NPs [56]. Of these, 8 genes are also found in our nNP gene group (*sfrp2*, *lrrn1*, *sox4*, *zic1*, *vim*, *rtn1*, *sox11*, *qk*), while 4 other genes (*khdrbs3*, *msi2*, *hrmtl3*, *tuba1*) show similar expression profile (up-regulated at day 3 NPs and/or day 8 rosettes) but were excluded due to the stringent criteria used to generate the clusters. Concerning the other 3 genes, one is mainly expressed in day 8 rosette NPs (*nhlh2*), another was not included in the microarrays (*Mm.156164*) and *slc2a1* is not differentially expressed during ES cell differentiation. The fact that none of the tNP genes were found in the embryonic Sox1-GFP^+^ population might be due to the limited number of genes screened in the embryo (384 in total) and to the expected transient character of tNPs *in vivo*, which might preclude their isolation from whole mouse embryos at E10.5. Nonetheless, this comparison reveals a strong correlation between the data generated from *in vitro* neural differentiation of ES cells and the *in vivo* data obtained from the developing mouse embryo, supporting our proposal that the gene signatures defining NP developmental stages *in vitro* might serve to identify similar stages during embryonic nervous system development.

The fifth group comprises genes that are up-regulated in day 8 cultures, when NPs are organized in neural tube-like rosettes and actively engaged in neurogenesis. Genes that were already up-regulated in day 3 NPs and that are linked to the previous stages of NP specification and proliferation (included in groups III and IV), were excluded from group V. In this way, this group is enriched in genes linked to the final stages of NP development and commitment to neuronal differentiation, revealing a gene expression profile in day 8 neural rosettes that matches the transcriptional landscape of the embryonic neural tube. For instance, proneural genes like *neurog1*, *neurog2* and *ascl1*, which are known to promote neuronal commitment, cell cycle exit and entry into differentiation of embryonic NPs, are included in group V. Additionally, genes encoding neuronal determination bHLH proteins, like *neurod4*, *nhlh1* and *nhlh2*, which are known to be activated by the proneural genes and function in early post-mitotic neurons to implement the neuronal differentiation program, are also present in this group. The similarities between embryonic neural tube and monolayer neural rosettes extend also to the increased transcription of genes of the Notch pathway, which are involved in regulating the balance between NP maintenance and differentiation, both in neural rosettes and in the embryonic neural tube. Other genes up-regulated in day 8 neural rosettes are known to be linked to neuronal type specification, like *lhx1*, *lhx9*, *islet1*, *lmo2* and various members of the Brn/Pou family, or associated with the general process of neuronal differentiation, like *dcx*, *elav1*, *2*, *3* and *4*, and *neurexin*. Altogether, this expression profile provides additional evidence, at the molecular level, of the similarities between the embryonic neural tube and the neural rosettes obtained by monolayer differentiation of ES cells.

A recent study reported the characterization of neural rosettes obtained by differentiating human ES cells through EBs or by co-culture with stromal cells [39]. Exposure of these rosettes to FGF2/EGF signalling resulted in the establishment of NS-like cells similar to those we obtained from mouse neural rosettes with the same growth factors. Gene expression profiling of these human ES cell-derived neural rosettes revealed a group of genes with highly increased expression in rosettes *vs.* human ES cells. Most of these are also highly expressed in the neural rosettes obtained from mouse ES cells described in this work (for instance, *plagl1*, *dach1*, *plzf/zbtb16*, *nr2f1*, *zic1*, *fabp7*, *lhx2*, *pou3f3*), suggesting a conserved general programme of NP/NSC development in mice and humans. Although these genes are highly expressed in rosette cells, our analysis reveals however that they are already up-regulated at day 3 of monolayer culture, before rosette formation, pointing to the existence of evolving populations of NPs/NSCs that emerge at different times of neural development.

To better define these NP populations, we took advantage of the simplicity of the monolayer method and the ability to purify Sox1-GFP^+^ NPs before rosette formation, to produce an accurate gene profiling dataset at various stages of *in vitro* neural development. By using embryo-oriented criteria to cluster the differentially expressed genes, our analysis did indeed allow us to pinpoint successive stages in the development of NPs, identified by unique gene signatures. A first signature defines a transient “tNP” population that emerges after neural induction and that gives rise to a subsequent population of “nNPs” with a different gene expression profile and already competent to enter neurogenesis. This is a “transit-amplifying” population of NPs that give rise to a final set of NPs organized in rosettes, expressing proneural genes and committed to exit the cell cycle and enter terminal differentiation. We propose that these stages also exist during embryonic development and future work shall explore whether the gene signatures here defined can serve to identify equivalent NP populations in the mouse embryo.

## Materials and Methods

### Maintenance and differentiation of mouse ES cells

The ES cell lines used for this study were E14tg2a and two derivatives, 46C (Sox1-GFP, [16]) and S25 (Sox2-βgeo, [15]), all three a gift from Meng Li (MRC Clinical Sciences Centre, Faculty of Medicine, Imperial College, London, UK) and Austin Smith (Wellcome Trust Centre for Stem Cell Research, University of Cambridge, Cambridge UK). ES cells were grown at 37°C in a 5% (v/v) CO_2_ incubator in Glasgow Modified Eagles Medium (GMEM, Invitrogen), supplemented with 10% (v/v) fetal bovine serum (FBS) (ES-qualified, Invitrogen), 2 ng/ml LIF and 1 mM 2-mercaptoethanol, on gelatin-coated (0.1% (v/v)) Nunc dishes. Cells were passaged every other day, at constant plating density of 3×10^4^ cells/cm^2^. To start the monolayer protocol, ES cells were plated in serum-free medium ESGRO Complete Clonal Grade medium (Millipore Inc.) at high density (1.5×10^5^ cells/cm^2^). After 24 hours, ES cells were gently dissociated and plated onto 0.1% (v/v) gelatin-coated tissue culture plastic at 1×10^4^ cells/cm^2^ in RHB-A or N2B27 media (StemCell Science Inc.), changing media every other day. For replating on day 4, cells were dissociated and plated at 2×10^4^ cells/cm^2^ onto laminin-coated tissue culture plastic in RHB-A medium supplemented with 5 ng/ml murine bFGF (Peprotech). From this point on, cells were replated in the same conditions every 4^th^ day and the medium was changed every 2^nd^ day, for the total of 20 days in culture. To quantify the number of differentiating neurons at each time point, cells were plated onto laminin-coated glass coverslips in 24-well Nunc plates and, 2 days after plating, medium was changed to a RHB-A:Neurobasal:B27 mixture (1∶1∶0.02), to allow a better survival of differentiated neurons. To obtain floating aggregates of NS cells, 3×10^5^ cells, dissociated at day 4, 8, 12, 16 and 20 of culture, were plated onto uncoated culture plastic in RHB-A medium supplemented with 10 ng/ml of recombinant murine EGF and bFGF (Peprotech) [57]. Floating aggregates formed within 24 hours and medium was changed after 48 h. After 4 days in suspension culture, aggregates were counted and plated *en bloc* onto laminin-coated coverslips, being then cultured for 4 days in RHB-A medium (with an intermediate medium change) to allow differentiation. When required, 10 µM BrdU (Sigma) was added to cultures for 5 min immediately before fixation.

### Treatment with γ-secretase inhibitor LY411575

Treatment with LY411575 was done at day 6, 10 or 14 after the beginning of the protocol. At these time points, culture medium was substituted by RHB-A: Neurobasal: B27 (1∶1∶0.02) medium supplemented either with 0.01% DMSO (control) or with 3 nM LY411575 (in 0.01% DMSO). Cells were fixed in 4% (w/v) paraformaldehyde after 24 h or 48 h of incubation, respectively, for the ISH and for the immunostaining.

### Immunocytochemistry

Fixed cells were blocked with 10% (v/v) FBS and 0.05% (v/v) Tween in phosphate buffered saline (PBS) for 1 hour, followed by incubation overnight with primary antibodies (Table S2). For all double immunostainings (with the exception of those with anti-GFP antibody), monolayer cultures of either S25 or E14tg2a ES cells were used. 46C cells were used in double immunostainings with anti-GFP antibody. Cells were washed 3 times in PBS followed by incubation for 1–2 hours with AlexaFluor-conjugated secondary antibodies (Molecular Probes) and DAPI (1∶10000, Sigma). For the detection of BrdU incorporation, cells were treated with 2N HCl for 30 min at 37°C at the beginning of the immunostaining procedure. Images of fixed cells were obtained with a DM5000B microscope and a DC350F camera (Leica Wetzlar, Germany). Living cells were photographed under an inverted microscope Leica DMIL with a DC200 camera. Images were processed by using Photoshop CS (Adobe, San Jose, CA).

The number of HuC/D and GFAP expressing cells was quantified as a proportion of the total number of cells in culture, counted with the help of ImageJ Cell Counter software. The number of positively labelled cells was quantified by counting 10 to 20 randomly selected fields per coverslip, corresponding to a minimum 5000 cells, counted as DAPI nuclei. Two coverslips were counted per each condition and the analysis was repeated for at least three independent experiments for each of S25 and 46C ES cell lines. Student t-test was used to compare means between groups and p-values lower than 0.05 were considered statistically significant.

### *In situ* hybridization

Digoxygenin-labeled RNA probes for *hes5*, *hes6* and *Dll1* were synthesized by T7 RNA polymerase from plasmid templates. Whole-mount ISH procedure [24] was adapted to cultured cells with minor modifications. After incubation with AP-conjugated anti-Dig antibody (Roche Diagnostics) coverslips containing cultured cells were incubated with AP substrate FastRed (Roche Diagnostics) for 0.5–1 h at 37°C. Anti-GFP immunostaining was performed after ISH when required.

### FACS analysis

Cells were dissociated and resuspended in 4% (v/v) FBS in PBS. Sox1-GFP analysis was performed on a FACS Calibur cytometer (Becton Dickinson), and all cell sorting experiments were done on a FACS Aria cell sorter (Becton Dickinson). Live cells were gated based on forward scatter and side scatter and/or by propidium iodide dye exclusion. For sorting, the GFP+ and GFP++ NPs populations were collected (the GFP negative cell fraction was discarded) and cell viability at the end of the FACS sorting procedure was determined using trypan blue dye exclusion method. FACS sorted cells were directly processed for RNA extraction.

### RNA extraction and RT-PCR

Total RNA was extracted from 10^6^ cells using High Pure RNA Isolation kit (Roche Diagnostics), with the inclusion of DNAseI treatment according to manufacturer's instructions. The first strand cDNA was synthesized from 0.5 µg of total RNA using SuperscriptII Reverse Transcriptase (Invitrogen) and random hexamers. After synthesis, each cDNA was diluted 5-fold and 5 µl of diluted cDNA used in PCR reaction with gene-specific primers (Table S3). The absence of contaminating genomic DNA was confirmed for each RNA extraction by PCR amplification of GAPDH-specific product from RT negative samples. The relative amount of each transcript was normalized to the level of GAPDH.

### Time-lapse movie

Day 4 or day 8 rosette NPs were plated onto laminin-coated MatTek dishes and rosettes were allowed to form for 24–48 h in a conventional CO_2_ incubator. Cultures were imaged on an inverted fluorescence Zeiss Axiovert 200M microscope in a chamber kept at 38°C. The chamber stage was buffered with 5% CO_2_/95% air mix and maintained in a humid environment. Images in bright field were captured using a 40×0.75 NA objective lens (Zeiss EC Plan-Neofluar) with the Hg-arc lamp and acquired with Metamorph software (Molecular Devices). The culture was permanently illuminated and seven focal points were imaged at 2 min intervals, for up to 16 hours. Data was analysed using ImageJ software, by choosing the most focused plane, adjusting brightness and contrast, and after instant time concatenation.

### Microarray sample preparation and data analysis

Total RNA was extracted from day 0 undifferentiated cells, day 1 ectodermal cells, day 3 FACS-purified Sox1-GFP+ and Sox1-GFP++ NPs, and day 8 neuroepithelial rosettes, using High Pure RNA Isolation kit (Roche Diagnostics). The preparation quality was assessed by agarose-formaldehyde gel electrophoresis. Three (or four in the case of 3++ samples) independent preparations (A to D), each containing total RNA from the day 0 (0), day 1 (1) day 3 (3+ and 3++) and day 8 (8) of differentiation were processed at the Max-Delbrück-Centrum für Molekulare Medizin (Berlin, Germany) according to the standardized procedures adopted by all members of the FunGenES European Consortium (http://www.fungenes.org/).

For the synthesis of double-stranded cDNA (from 15 µg of total RNA) the cDNA synthesis system kit (Roche Diagnostics) was used. Biotinylated cRNA were synthesized with Perkin-Elmer nucleotide analogues using the Ambion MEGAScript T7 kit. After fragmenting of the cRNA for target preparation using the standard Affymetrix protocol, 15 µg fragmented cRNA were hybridized for 16 h at 45°C to Mouse Genome 430 Version 2.0 Array (Affymetrix) which includes 45101 probe sets. Following hybridization, arrays were washed and stained with streptavidin-phycoerythrin in the Affymetrix Fluidics Station 450 and further scanned using the Affymetrix GeneChip Scanner 3000 7G. The image data were analyzed with GCOS 1.4 using Affymetrix default analysis settings and global scaling as normalization method. All chips passed quality criteria. Microarray data reported in the manuscript is described in accordance with MIAME guidelines and original datasets have been deposited in the ArrayExpress database for open access (Accession Number E-TABM-717).

After RMA normalization [31], a parametric ANOVA (F-test) and ten pair-wise comparisons using the Student t-test (unpaired, assuming unequal variances) were performed for each time point independently. The false discovery rate of each test-set was calculated using the Benjamini Hochberg procedure [58]. Finally, an ANOVA FDR-value<10^−3^ was used to identify and restrict the number of differentially expressed probe sets (n = 9456). This corresponds to a total of 6563 genes.

To cluster these genes in groups with similar expression profiles along the selected four time points of the monolayer protocol, a cut-off value of 2 for the fold differences in expression levels between time points was imposed. Five groups were defined according to the following criteria (Table S1): I. “ES cells group”–Expression level on day 0 is at least twice higher than expression in all other time points (days 1, 3 and 8); II. “PE group”–Expression level on day 1 is at least twice higher than expression in all other time points (days 0, 3 and 8); III. “tNPs group”–Expression level on day 3 is at least twice higher than expression in all other time points (days 0, 1 and 8); IV. “nNPs group”–Expression level on day 3 (3+ and/or 3++) is at least twice higher than expression in earlier time points (days 0 and 1), with expression level at day 8 being equal or higher than at day 3 (day (3++)); V. “Rosette group” - Expression level on day 8 is at least twice higher than expression in all other time points (days 0, 1 and 3); in addition, expression levels at day 0, 1 and 3 cannot increase more than twice between them.

## Supporting Information

Figure S1 Expression of nanog and Fgf5 at successive time points of rosette cultures, using RT-PCR
(0.09 MB TIF)

Figure S2 Expression of BMP pathway genes obtained by microarray analysis. Fold changes, relative to day 0, obtained from Affymetrix profiling for the genes encoding BMP agonists bmp4 and nodal, and BMP inhibitors chordin-like1 (chrld1) and follistatin (fstl1).
(0.08 MB TIF)

Figure S3 Expression of Notch pathway genes obtained by microarray analysis. Fold changes, relative to day 0, obtained from Affymetrix profiling for the genes encoding Notch receptors Notch1 and Notch2, Notch ligands Dll1 and Dll3, and Notch targets hes5 and hes6.
(1.83 MB TIF)

Table S1 Affymetrix profiling data. A) Worksheet All: list of all probe sets and respective symbols, title, physical position and ANOVA:values. B) Worksheet 9456 probe sets: list of 9456 probe sets (6563 genes), and respective symbols and title, with ANOVA values lower than 10∶3. C) Worksheet ES cells: list of 226 probe sets (188 genes) that belong to the ES cell expression group. D) Worksheet PE: list of 80 probe sets (66 genes) that belong to the PE expression group. E) Worksheet tNPs: list of 75 probe sets (61 genes) that belong to the tNPs expression group. F) Worksheet nNPs: list of 1171 probe sets (763 genes) that belong to the nNPs expression group. G) Worksheet Rosettes: list of 919 probe sets (673 genes) that belong to the Rosette expression group.
(8.77 MB XLS)

Table S2 List of antibodies used for the immunostaining analyses
(0.04 MB DOC)

Table S3 List of gene-specific primers used in RT-PCR
(0.13 MB DOC)

Movie S1 Interkinetic nuclear movement (INM) in rosette cultures. Day 6 rosette NPs imaged on an inverted fluorescence Zeiss Axiovert 200M microscope. The culture was permanently illuminated and seven focal points were imaged at 2 min intervals, for up to 16 hours. Data were analysed using ImageJ software, by choosing the most focused plane, adjusting brightness and contrast, and after instant time concatenation.
(9.56 MB ZIP)
