# Supplementary figures and images for: Neural Differentiation of Embryonic Stem Cells In Vitro: A Road Map to Neurogenesis in the Embryo
Source: PLoS One. 2009 Jul 21;4(7):e6286. doi: 10.1371/journal.pone.0006286 (PMC2709448; doi:10.1371/journal.pone.0006286)

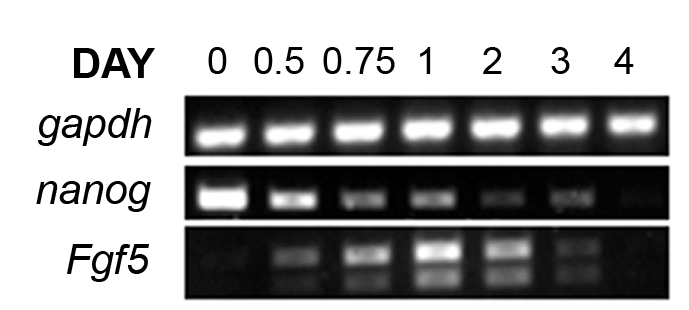

Supplement: Figure S1 — Expression of nanog and Fgf5 at successive time points of rosette cultures, using RT-PCR (0.09 MB TIF) [file pone.0006286.s001.tif]

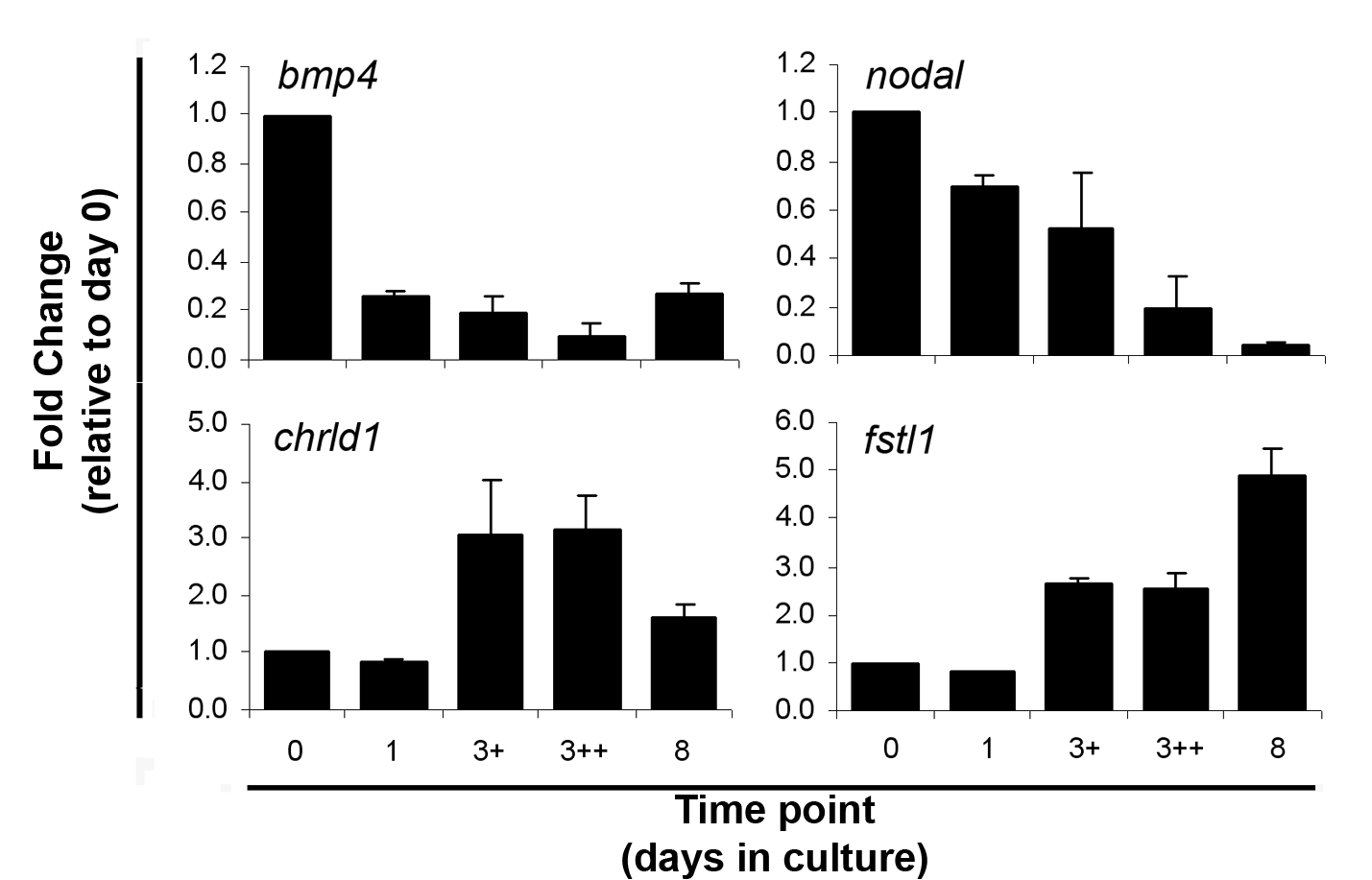

Supplement: Figure S2 — Expression of BMP pathway genes obtained by microarray analysis. Fold changes, relative to day 0, obtained from Affymetrix profiling for the genes encoding BMP agonists bmp4 and nodal, and BMP inhibitors chordin-like1 (chrld1) and follistatin (fstl1). (0.08 MB TIF) [file pone.0006286.s002.tif]

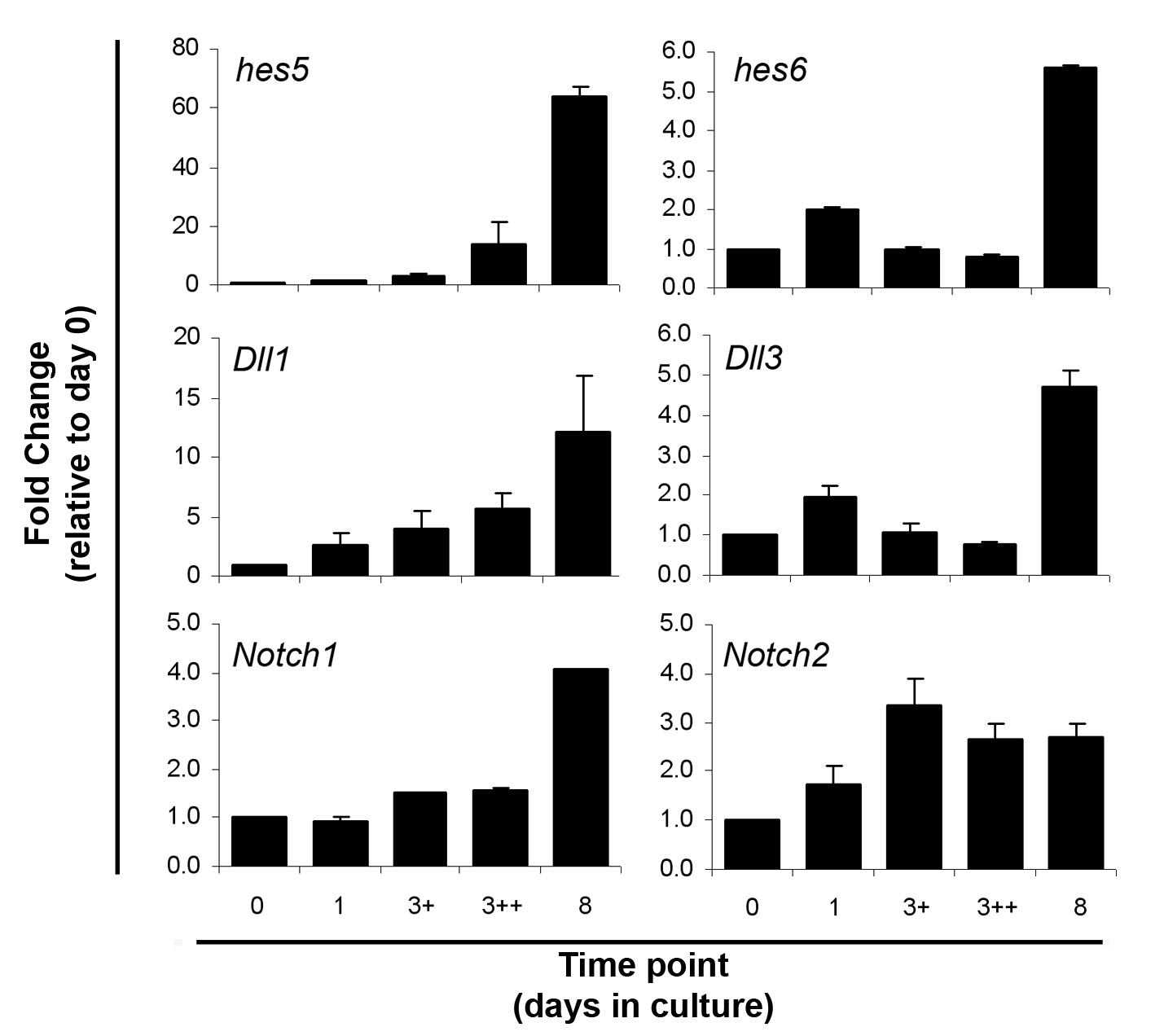

Supplement: Figure S3 — Expression of Notch pathway genes obtained by microarray analysis. Fold changes, relative to day 0, obtained from Affymetrix profiling for the genes encoding Notch receptors Notch1 and Notch2, Notch ligands Dll1 and Dll3, and Notch targets hes5 and hes6. (1.83 MB TIF) [file pone.0006286.s003.tif]
