# Supplementary material for: Neural Differentiation of Embryonic Stem Cells In Vitro: A Road Map to Neurogenesis in the Embryo
Source: PLoS One. 2009 Jul 21;4(7):e6286. doi: 10.1371/journal.pone.0006286 (PMC2709448; doi:10.1371/journal.pone.0006286)
Supplement: Table S2 — List of antibodies used for the immunostaining analyses (0.04 MB DOC) [file pone.0006286.s005.doc]

| **Antibody anti-…** | **Dilution** | **Animal** | **Origin** |
| --- | --- | --- | --- |
| Afadin | 1/500 | Rabbit | Sigma #A0224 |
| aPKCζ (C-20) | 1/200 | Rabbit | Santa Cruz #sc-216 |
| PAR3 | 1/1500 | Rabbit | Upstate Biotech #07-330 |
| b-catenin | 1/1000 | Rabbit | Sigma #C2206 |
| Doublecortin (DCX) | 1/3000 | Guinea pig | Chemicon #AB5910 |
| BrdU cl. BU 33 | 1/1000 | Mouse | Sigma #B2531 |
| E-Cadherin | 1/100 | Mouse | BD Transduction Lab. #610181 |
| GFAP, Cl G-4-5 | 1/400 | Mouse | Sigma #G3893 |
| GFP | 1/500 | Rabbit | Abcam #ab290 |
| GLAST | 1/4000 | Guinea pig | Chemicon |
| gamma-Tubulin | 1/500 | Rabbit | Sigma #T3559 |
| HuC/D | 1/500 | Mouse | Molecular Probes #A-21271 |
| N-Cadherin | 1/200 | Mouse | BD Transduction Lab. #610920 |
| Numb | 1/50 | Mouse | DSHB (Cmnb-1) |
| O4 | 1/100 | Mouse | Chemicon #MAB345 |
| Phospho-Histone H3 | 1/200 | Rabbit | Upstate Biotech #06-570 |
| Sox1 | 1/100 | Rabbit | Sigma #S8318 |
| Sox2 | 1/200 | Rabbit | Chemicon #AB5603 |
| Tuj1 | 1/500 | Mouse | Covance # MMS-435P |
| ZO-1 | 1/50 | Mouse | Zymed #33-9100 |

**Supplementary Table 2.** List of antibodies used for the immunostaining analyses.
