# Supplementary material for: Neural Differentiation of Embryonic Stem Cells In Vitro: A Road Map to Neurogenesis in the Embryo
Source: PLoS One. 2009 Jul 21;4(7):e6286. doi: 10.1371/journal.pone.0006286 (PMC2709448; doi:10.1371/journal.pone.0006286)
Supplement: Table S3 — List of gene-specific primers used in RT-PCR (0.13 MB DOC) [file pone.0006286.s006.doc]

| **Gene** | **Reference** | **T annealing** | **Fragment (bp)** | **Forward Primer (5’ – 3’)** | **Reverse Primer (5’ – 3’)** | **# cycles** |
| --- | --- | --- | --- | --- | --- | --- |
| **BLBP (fabp7)** | this paper | 58º | 213 | GGGTAAGACCCGAGTTCCTC | ATCACCACTTTGCCACCTTC | 30 |
| **Dll1** | this paper | 64º | 510 | ACTCCTTCAGCCTGCCTGA | TATCGGATGCACTCATCGC | 35 |
| **Dll3** | this paper | 58º | 219 | CAAGACGGTGCTGGGGATGG | CGGTAGGGGGAGGTAGAGAT | 35 |
| **Emx2** | [1] | 60º | 386 | GTTTATGTGATCCCCGCACT | GTGGGGGTAAGGAAAGGAAG | 35 |
| **FGF5** | this paper | 60º | 394 | CTTCTGCCTCCTCACCAGTC | CACTCTCGGCCTGTCTTTTC | 35 |
| **FoxG1 (BF1)** | this paper | 60º | 438 | CTGACGCTCAATGGCATCTA | TTTGAGTCAACACGGAGCTG | 35 |
| **GAPDH** | this paper | 60º | 466 | ATTCAACGGCACAGTCAAGG | TGGATGCAGGGATGATGTTC | 28 |
| **GFAP** | [2] | 62º | 207 | CCAAACTGGCTGATGTCTACC | GCTTCATGTGCCTCCTGTCTA | 35 |
| **GLAST(slc1a3)** | [1] | 58º | 230 | CCAAAAGCAACGGAGAAGAG | CCTCCCGGTAGCTCATTTTA | 32 |
| **Hes1** | this paper | 60º | 376 | AAAGACGGCCTCTGAGCACA | TCATGGCGTTGATCTGGGTCA | 35 |
| **Hes3** | this paper | 60º | 244 | GATACGGAAACGAAAGCTGG | GTGGGTTGGCGCTGTCCGTG | 35 |
| **Hes5** | this paper | 60º | 353 | AAGTACCGTGGCGGTGGAGAT | CGCTGGAAGTGGTAAAGCAGC | 35 |
| **Hes6** | this paper | 60º | 204 | CTCCCTCGTGTTCACCTCTC | GAGGAGCAGCTTCAGTGACC | 32 |
| **Hoxb4** | [3] | 60º | 260 | CCTGGATGCGCAAAGTTC | GTGTTGGGCAACTTGTGGTC | 35 |
| **Hoxb9** | [3] | 60º | 182 | GGAAGCGAGGACAAAGAGAG | TTGAGGAGTCTGGCCACTTC | 35 |
| **Jagged 1** | this paper | 60º | 397 | CCAGCCAGTGAAGACCAAGT | TCAGCAGAGGAACCAGGAAA | 32 |
| **Jagged 2** | this paper | 58º | 119 | GAGGTCAAGGTGGAAACAGT | TGTCCACCATACGCAGATAA | 35 |
| **Mash1** | this paper | 60º | 137 | AGATGAGCAAGGTGGAGACG | TGGAGTAGTTGGGGGAGATG | 35 |
| **Math1** | [4] | 62º | 185 | CTTCCTCTGGGGGTTACTCG | AAACTCTCCGTCACTTCTGTGG | 35 |
| **Nanog** | [5] | 57º | 464 | ATGAAGTGCAAGCGGTGGCAGAAA | CCTGGTGGAGTCACAGAGTAGTTC | 32 |
| **Nestin** | [6] | 65º | 403 | CTGGAACAGAGATTGGAAGGCCGCT | GGATCCTGTGTCTTCAGAAAGGCTGTCAC | 30 |
| **Ngn1** | this paper | 60º | 319 | ATGCCTGCCCCTTTGGAGAC | TGCATGCGGTTGCGCTCGC | 35 |
| **Ngn2** | this paper | 60º | 342 | GCTGGCATCTGCTCTATTCC | ATGAAGCAATCCTCCCTCCT | 35 |
| **Notch1** | this paper | 55º | 126 | CGGTGAACAATGTGGATGCT | ACTTTGGCAGTCTCATAGCT | 32 |
| **Notch2** | this paper | 55º | 242 | GTGGAGGCGACTCTTCTGCT | GCTGGGAGTCACGTTATACT | 32 |
| **Notch3** | this paper | 55º | 166 | GAGGCTACCTTGGCTCTGCT | GGCAGCCTGTCCAAGTGATCT | 32 |
| **Oct4** | this paper | 62º | 484 | CTGAGGGCCAGGCAGGAGCACGAG | CTGTAGGGAGGGCTTCGGGCACTT | 30 |
| **Olig2** | this paper | 62º | 144 | CACAGGAGGGACTGTGTCCT | GGTGCTGGAGGAAGATGACT | 30 |
| **Otx2** | [7] | 65º | 347 | ACAAGTGGCCAGTTCAGTCC | CTGGGTGGAAAGAGAAGCTG | 35 |
| **Pax6** | this paper | 60º | 249 | AGGGGGAGAGAACACCAACT | CATTTGGCCCTTCGATTAGA | 32 |
| **Sox1** | this paper | 60º | 381 | CCAAGAGACTGCGCGCGCTG | GGGTGCGCCGGGTGTGCGTG | 35 |
| **Sox2** | [5] | 65º | 192 | ATGGACAGCTACGCGCAC | CGAGCCGTTCATGTAGGTCTG | 35 |
| **Sox21** | this paper | 60º | 170 | ATGGGCAAGCCTCAGCTGGA | CGCCTCTGCCTGACACCTA | 35 |
| **Sox3** | this paper | 65º | 209 | CAGGCAACGGGGGCAGCGGG | CCGCATCGGTCAGCAGTTTC | 35 |
| **T(Brachyury)** | [5] | 58º | 645? | ATGCCAAAGAAAGAAACGAC | AGAGGCTGTAGAACATGATT | 35 |
| **Tau** | this paper | 65º | 268 | CTTTGAACCAGTATGGCTGACCCT | CGAGGTGTGGCGATCTTCG | 30 |
| **TujI** | [2] | 62º | 208 | AAGGTAGCCGTGTGTGACATC | ACCAGGTCATTCATGTTGCTC | 28 |

**Supplementary Table 3.** List of gene-specific primers used in RT-PCR.

**References:**

1. Conti L, Pollard SM, Gorba T, Reitano E, Toselli M, et al. (2005) Niche-Independent Symmetrical Self-Renewal of a Mammalian Tissue Stem Cell. PLoS Biology 3: 1594-1606.

2. Ahn J, Lee K, Shin D, Shim J, Kim C, et al. (2004) Temporal expression changes during differentiation of neural stem cells derived from mouse embryonic stem cell. J Cell Biochem 93: 563-578.

3. Chiba S, Kurokawa M, Yoshikawa H, Ikeda R, Takeno M, et al. (2005) Noggin and basic FGF were implicated in forebrain fate and caudal fate, respectively, of the neural tube-like structures emerging in mouse ES cell culture. Experimental brain research 163: 86-99.

4. Mizuseki K, Sakamoto T, Watanabe K, Muguruma K, Ikeya M, et al. (2003) Generation of neural crest-derived peripheral neurons and floor plate cells from mouse and primate embryonic stem cells. PNAS 100: 5828-5833.

5. Ying QL, Nichols J, Chambers I, Smith A (2003) BMP induction of Id proteins suppresses differentiation and sustains embryonic stem cell self-renewal in collaboration with STAT3. Cell 115: 281-292.

6. Abe Y, Kouyama K, Tomita T, Tomita Y, Ban N, et al. (2003) Analysis of neurons created from wild-type and Alzheimer's mutation knock-in embryonic stem cells by a highly efficient differentiation protocol. J Neurosci 23: 8513-8525.

7. Smukler SR, Runciman SB, Xu S, van der Kooy D (2006) Embryonic stem cells assume a primitive neural stem cell fate in the absence of extrinsic influences. J Cell Biol 172: 79-90.
